# Fe_2_O_3_ Magnetic Nanoparticles and Curcumin Improved Sperm Parameters in Rats with Scrotal Hyperthermia

**DOI:** 10.31661/gmj.v10i0.2014

**Published:** 2022-02-02

**Authors:** Maryam Mollaei, Mehrdad Hashemi, Elham Siasi, Sayeh Jafari Marandi, Malihe Entezari

**Affiliations:** ^1^Department of Genetics, Faculty of biosciences, Islamic Azad University, North Tehran Branch, Tehran, Iran; ^2^Department of Genetics, Faculty of Advanced Science and Technology, Islamic Azad University, Tehran, Iran; ^3^Farhikhtegan Medical Convergence Sciences Research Center, Farhikhtegan Hospital Tehran Medical Sciences, Islamic Azad University, Tehran, Iran

**Keywords:** Sperms, Rats, Fe_2_O_3_, Scrotal Hyperthermia

## Abstract

**Background:**

Testicular function depends on temperature, and it has been shown that scrotal hyperthermia causes a sharp decrease in sperm parameters due to oxidative stress. In recent years, the use of natural materials from the plant and nanoparticles has attracted much attention. Therefore, the present study aimed to investigate the effect of curcumin and Fe_2_O_3_ nanoparticles on sperm parameters in rats.

**Materials and Methods:**

After preparing the rats, they were placed in a hot water bath at 43°C for 30 minutes for six consecutive days. The 48 rats were then divided into eight groups. A concentration of 0.03 mg/kg body weight magnetic Fe_2_O_3_ nanoparticles and curcumin at the concentration of 0.02 mg/kg body weight were used. After killing animals, the semen parameters such as viability, concentration, motility, and morphology of sperm were studied.

**Results:**

Significant differences were observed in all groups of rats in terms of semen parameters (P<0.001). The results showed a positive effect of curcumin on improving semen parameters in scrotal hyperthermia rats and a negative and toxic effect of Fe_2_O_3_ magnetic nanoparticles. However, significant improvement in sperm parameters was observed when Fe_2_O_3_ magnetic nanoparticles were given to rats along with curcumin.

**Conclusion:**

Curcumin has a positive and significant effect on improving sperm parameters in scrotal hyperthermia conditions. Fe_2_O_3_ magnetic nanoparticles, if co-administered with curcumin, can significantly improve sperm parameters. In this regard, green synthesis of nanoparticles and concomitant administration of antioxidants such as curcumin in scrotal hyperthermia conditions is recommended.

## Introduction

Spermatogenesis is a multi-step, complex process in which mature sperm are produced following the proliferation and differentiation of spermatogonia cells. The spermatogonia and the process of spermatogenesis are in the testicles, and in most mammals, they must be outside the body cavity at a temperature of 2 to 8°C below the body temperature to perform best [1]. Because spermatogenesis is a temperature-dependent process, increasing testicular temperature disrupts this process [2].Several studies have reported adverse and destructive effects of scrotal hyperthermia, or increased testicular temperature, on sperm parameters and spermatogenesis processes in various species, including humans, mice, rats, sheep, pigs, and cows [3].Researchers have shown that scrotal hyperthermia, both transient and persistent, can cause serious damage to the spermatogenesis process [4]. The findings also show that thermal shock affects sperm quality and reduces sperm motility and fertilization of ovule and sperm [5].As the studies show, increasing the temperature is a stress for the living cell, and they can respond to this stress through hypoxia stress pathways, oxidative stress, and apoptosis [2].Findings have shown that following the hypoxia response, mitochondria are the main site of production of reactive oxygen species [6]. In general, it can be stated that oxidative stress plays an important role in causing hyperthermia-induced abnormalities in sperm parameters. Reactive oxygen species (ROS) have a high affinity, so it reacts with all cellular components, including DNA, lipids, and proteins, which are the main targets for attack by these species.As a result of the interaction of free radicals with the cellular genome, a variety of physiological disorders and eventually cell death occurs during cascading events [7].Because the spermatogenesis process is very active and it is estimated that 1,000 sperm are produced per second, a high rate of cell division occurs, indicating the use of mitochondrial oxygen by the germinal epithelium [8].These tissues are very sensitive to ROS-induced oxidative stress [9]. Curcumin is an active ingredient in the turmeric plant that has antioxidant, anti-inflammatory, and anti-diabetic properties and reduces fat, glucose, and cholesterol [10].Curcumin has unique antioxidant properties by having two antioxidant properties, including phenolic rings and diketone moiety on a molecule [11]. The use of curcumin in the diet of rats treated with heavy metals, including Lead and Cadmium has been reported to protect the testicular structure, improve the number of reproductive cells, and spermatogenesis by reducing oxidative stress and scavenging free radicals [12,13]. Curcumin also modulates testicular structure and spermatogenesis in mice treated with ultraviolet irradiation [14].pid metabolism of the curcumin, which, due to its low bioavailability, limits its use [15]. Nanotechnology is the production and control of materials in dimensions between 1-100nm. Synthetic nanomaterials are widely used in medicine, biotechnology, agriculture, etc. [1]. Following recent advances in nanotechnology, metal oxide nanoparticles can be used in various fields, from light-electron materials to sensors, environmental remediation, and biomedical medicine [2].Despite its many benefits, nanoparticles can be associated with responses such as chronic inflammation and the production of oxygen-free radicals. One of the most important metal oxide nanoparticles that have been considered in recent years is magnetic Fe_2_O_3_ nanoparticles [16]. The excellent properties of these nanoparticles include their fast effect, high magnetic properties, and small size, which has led to their many applications in various fields [17,18].Due to extensive research on the widespread use of Fe_2_O_3_ magnetic nanoparticles such as tumor treatment, magnetic resonance imaging, drug delivery, and gene transfer to tissues and cells, there are little reports on their side effects on testicular cells. Also, the effect of concomitant administration of Fe_2_O_3_ nanoparticles with curcumin antioxidants in patients with sclerotic hyperthermia has not been studied.Therefore, the present study aimed to investigate the effect of Fe_2_O_3_ magnetic nanoparticles with curcumin on sperm parameters in rats with scrotal hyperthermia.

## Materials and Methods

### Materials

FeO nanoparticles at the concentration of 5mg/mL were purchased from Sigma Aldrich (Germany) with a purity of more than 97% and particle size of 5 nm. Curcumin was purchased from Merck Cop (Germany) with a purity of more than 80% and a molecular weight of 388.38.The dose of FeO nanoparticles was determined based on LD50, namely, the concentration that caused the death of half of the rats. Accordingly, concentrations (0.005, 0.01, 0.02, 0.03, 0.04 and 0.05 mg/kg body weight [BW]) were given to the rats and LD50 was determined as 0.02 mg/kg BW. Therefore, this concentration was used in subsequent experiments.

### Animals

Forty-eight adult male rats was purchased from the Pasteur Institute (Tehran, Iran). The animals were kept under standard conditions of 12 hours of light and 12 hours of darkness 25±2°C and relative humidity of 50%±10%. All animals were fed the same proportions of corn, wheat, barley, and pellets under the same nutritional conditions, and free access to water was available to all.

### Induction of Scrotal Hyperthermia

Scrotal hyperthermia was induced by placing a scrota-containing testicles in a hot water bath (Memmert, Germany) at 43°C for 30 minutes once a day for six consecutive days. The control rats were placed in a water bath at 22°C. After induction of scrotal hyperthermia, the animals were dried and examined for any damage on the scrota and then placed in cages. Studies have shown that no animals were harmed.After scrotal hyperthermia induction, the rats were randomly divided into eight groups as follow:1. Control group2. Control group receiving magnetic FeO nanoparticle (0.03 mg/kg BW) 3. Control group receiving curcumin (0.02 mg/kg BW)4. Control group receiving magnetic FeO nanoparticles (0.03 mg/kg BW) and curcumin (0.02 mg/kg BW) simultaneously5. Scrotal hyperthermia group6. Scrotal hyperthermia group receiving FeO nanoparticle (0.03 mg/kg BW)7. Scrotal hyperthermia group receiving curcumin (0.02 mg/kg BW)8. Scrotal hyperthermia group receiving FeO nanoparticles (0.03 mg/kg BW) and curcumin (0.02 mg/kg BW) simultaneouslyAfter completing the treatments, all the animals were killed by an overdose of anesthesia, and then the testicular tissue was removed for tissue tests and the semen collected from the epididim for sperm analysis and cellular examination parameters.

### Sperm Analysis

In order to analyze sperm, four attributes, including morphology, viability, concentration, viability, and motility of sperm were evaluated.So we first collected the semen from the epididymal tissue. Sperm (10 μL) were transferred to a hemocytometer (Z359629, Merck, Germany), and sperm counts were performed under an optical microscope (BM180N, Novel, China) with a magnification of 40X.Sperm motility was evaluated by a microscope in ten fields based the World Health Organization recommendation.Sperm 3-(4, 5-dimethylthiazol-2-yl)-2, 5-diphenyltetrazolium bromide (MTT; Sigma, USA) viability assay introduced by Nasr-Esfahani (2002) was used to evaluate sperm viability [19].Alanine blue staining (Sigma, USA)was also used to study sperm morphology. The slides were evaluated for morphological disorders in the tail, neck or head.

### Ethical Issues

This study was approved by Azad University, Tehran Shomal Branch, Tehran, Iran by the ethics code of IR.IAU.TNB.REC.1399.001.

### Statistical Analysis

One-way analysis of variance (ANOVA) was used to identify significant differences in the studied characteristics among the rats groups. SPSS software (version 22, IBM, USA) was used to analyze the data. P<0.05 was considered as statistically significant.

## Results

**Figure 1:**
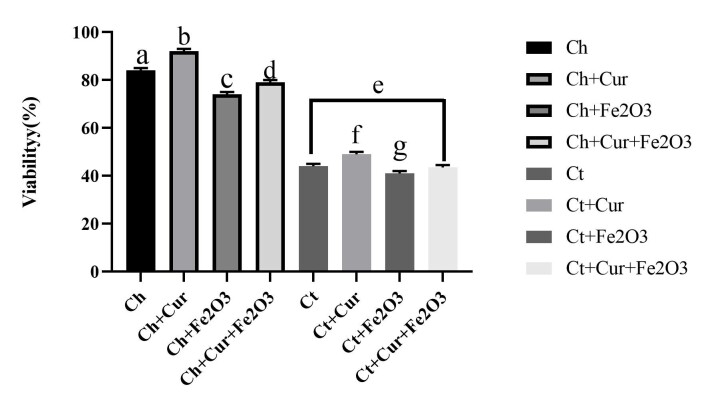
The effect of curcumin and Fe_2_O_3_ nanoparticles on sperm viability percentage in different rat groups. **Ch:** Healthy control; **Ch+Cur:** Healthy control receiving curcumin; **Ch+Fe_2_O_3_ :** Healthy control receiving Fe_2_O_3_ nanoparticles; **Ch+Cur+Fe_2_O_3_ :** Healthy control receiving curcumin and Fe_2_O_3_ nanoparticles; **Ct:** Rats with scrotal hyperthermia; **Ct+cur:** Rats with scrotal hyperthermia receiving curcumin; **Ct+ Fe_2_O_3_ :**Rats with scrotal hyperthermia receiving Fe_2_O_3_ nanoparticles; **Ct+Cur+ Fe_2_O_3_ :** Rats with scrotal hyperthermia receiving curcumin and Fe_2_O_3_ nanoparticles. The different letters indicate significant differences between the groups according to the Tukey test atthe probability level of 0.001.

**Figure 2:**
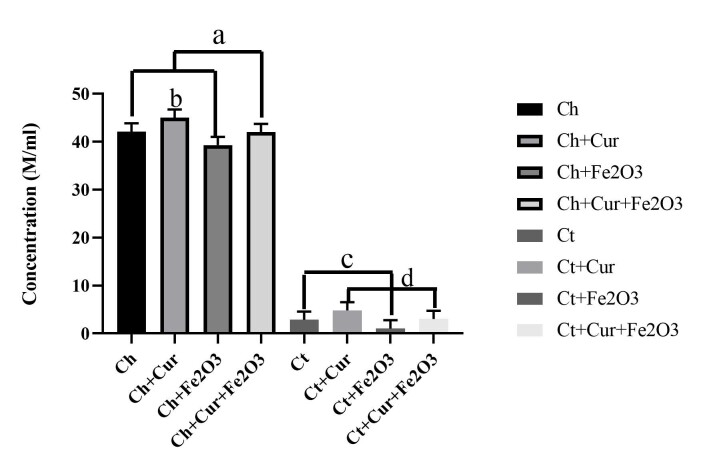
The effect of curcumin and Fe_2_O_3_ nanoparticles on sperm concentration in different rat groups. **Ch:** Healthy control; **Ch+Cur:** Healthy control receiving curcumin; **Ch+Fe_2_O_3_ :** Healthy control receiving Fe_2_O_3_ nanoparticles; **Ch+Cur+Fe_2_O_3_ :** Healthy control receiving curcumin and Fe_2_O_3_ nanoparticles; **Ct:** Rats with scrotal hyperthermia; **Ct+cur:** Rats with scrotal hyperthermia receiving curcumin; **Ct+ Fe_2_O_3_ :**Rats with scrotal hyperthermia receiving Fe_2_O_3_ nanoparticles; **Ct+Cur+ Fe_2_O_3_ :** Rats with scrotal hyperthermia receiving curcumin and Fe_2_O_3_ nanoparticles. The different letters indicate significant differences between the groups according to the Tukey test atthe probability level of 0.001.

**Figure 3:**
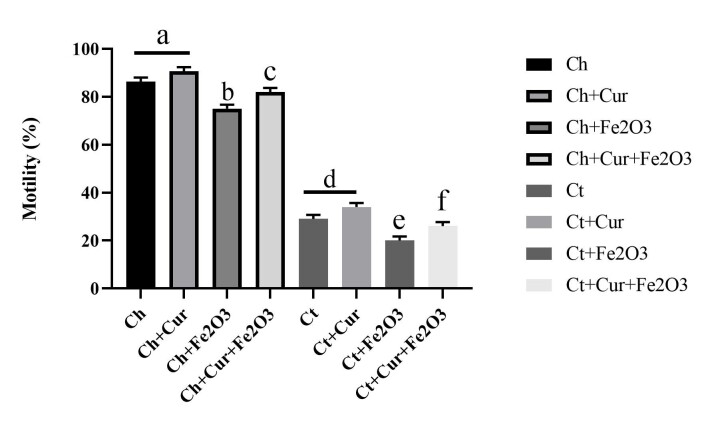
The effect of curcumin and Fe_2_O_3_ nanoparticles on sperm motility percentage in different rat groups. **Ch:** Healthy control; **Ch+Cur:** Healthy control receiving curcumin; **Ch+Fe_2_O_3_ :** Healthy control receiving Fe_2_O_3_ nanoparticles; **Ch+Cur+Fe_2_O_3_ :** Healthy control receiving curcumin and Fe_2_O_3_ nanoparticles; **Ct:** Rats with scrotal hyperthermia; **Ct+cur:** Rats with scrotal hyperthermia receiving curcumin; **Ct+ Fe_2_O_3_ :**Rats with scrotal hyperthermia receiving Fe_2_O_3_ nanoparticles; **Ct+Cur+ Fe_2_O_3_ :** Rats with scrotal hyperthermia receiving curcumin and Fe_2_O_3_ nanoparticles. The different letters indicate significant differences between the groups according to the Tukey test atthe probability level of 0.001.

**Figure 4:**
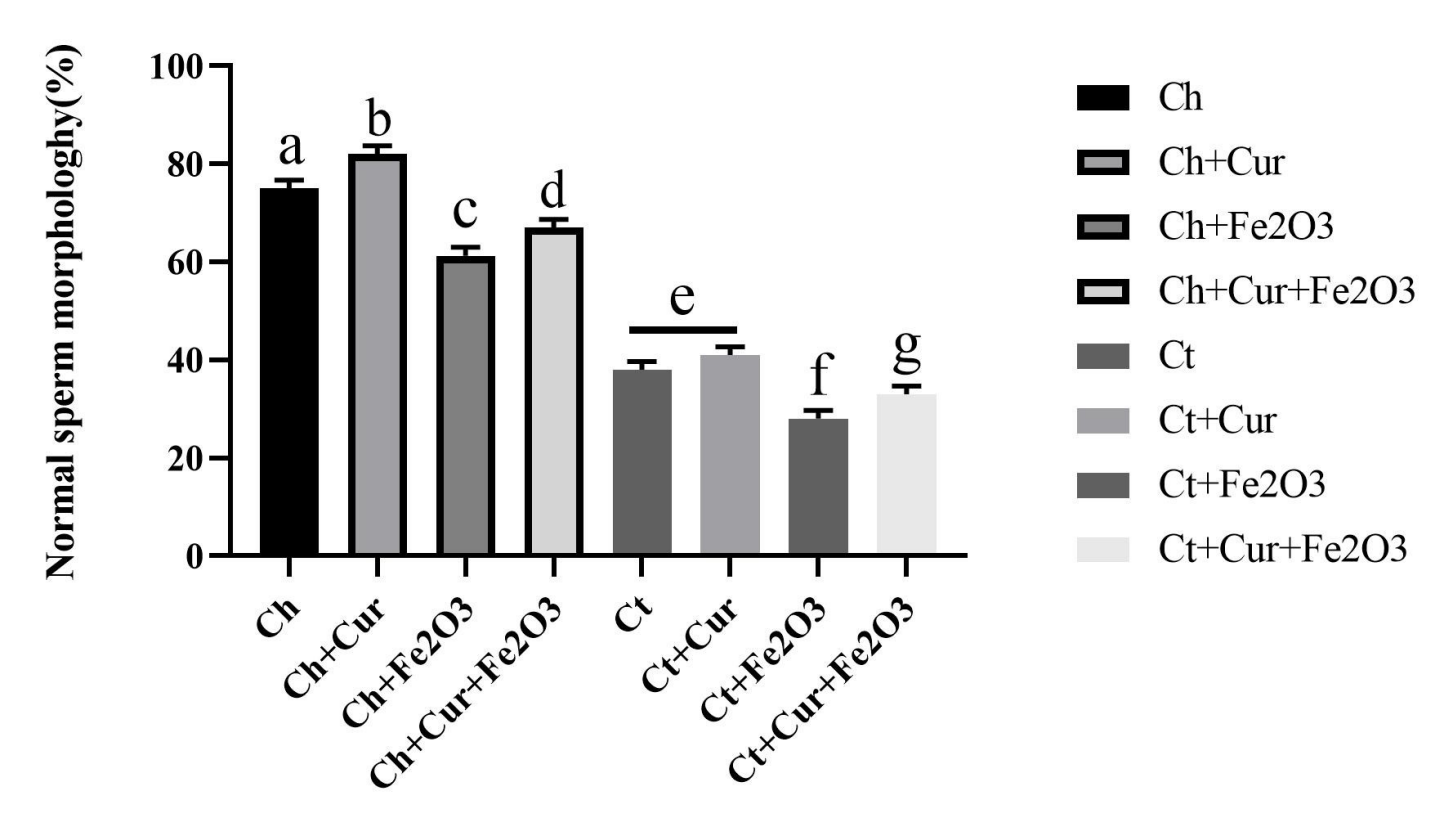
The effect of curcumin and Fe_2_O_3_ nanoparticles on the percentage of sperms with normal morphology in different rat groups. **Ch:** Healthy control; **Ch+Cur:** Healthy control receiving curcumin; **Ch+Fe_2_O_3_ :** Healthy control receiving Fe_2_O_3_ nanoparticles; **Ch+Cur+Fe_2_O_3_ :** Healthy control receiving curcumin and Fe_2_O_3_ nanoparticles; **Ct:** Rats with scrotal hyperthermia; **Ct+cur:** Rats with scrotal hyperthermia receiving curcumin; **Ct+ Fe_2_O_3_ :**Rats with scrotal hyperthermia receiving Fe_2_O_3_ nanoparticles; **Ct+Cur+ Fe_2_O_3_ :** Rats with scrotal hyperthermia receiving curcumin and Fe_2_O_3_ nanoparticles. The different letters indicate significant differences between the groups according to the Tukey test atthe probability level of 0.001.

Sperm Viability Percentage The results of the present study showed that sperm viability was reduced by the induction of scrotal hyperthermia. In healthy rats, the addition of curcumin had no significant effect on sperm viability, but Fe_2_O_3_ magnetic nanoparticles reduced sperm viability. However, in rats with scrotal hyperthermia, curcumin increased sperm viability. In the present study, the positive effect of concomitant administration of F2O3 magnetic nanoparticles and curcumin on sperm viability was observed. However, the lowest sperm viability was observed in rats with scrotal hyperthermia treated with Fe_2_O_3_ magnetic nanoparticles (Figure-1). Sperm Concentration The present study results indicated that there were significant differences in sperm concentration in semen between different study groups (P<0.001). The highest concentrations of sperm were observed in the healthy rats group receiving curcumin and the concomitant recipient of Fe_2_O_3_ magnetic nanoparticles with curcumin. However, in healthy rats, administration of Fe_2_O_3_ nanoparticles significantly reduced sperm concentration. In rats with Scrotal Hyperthermia, sperm concentration was significantly reduced. The lowest sperm concentrations were observed in rats with Scrotal Hyperthermia receiving Fe_2_O_3_ magnetic nanoparticles. However, administration of curcumin or administration of Fe_2_O_3_ nanoparticles in combination with curcumin significantly increased sperm concentration compared with control (Figure-2). Sperm Motility Significant differences in sperm motility were observed in different study groups (P<0.001). The results of the current study showed that scrotal hyperthermia greatly reduces sperm motility. In the current study, healthy rats and healthy rats receiving curcumin had the highest sperm motility. In healthy rats, the lowest sperm motility was obtained in the rats receiving Fe_2_O_3_ nanoparticles. However, in healthy rats, concomitant administration of Fe_2_O_3_ magnetic nanoparticles along with curcumin led to a significant increase in sperm motility. In scrotal hyperthermia rats, the lowest sperm motility was reported in Fe_2_O_3_ nanoparticle recipient rats. However, in the rats with scrotal hyperthermia, curcumin or concomitant administration of Fe_2_O_3_ magnetic nanoparticles along with curcumin, higher sperm motility was observed compared with the control group (Figure-3). Sperm Morphology The results of the present study showed a positive and significant effect of curcumin on increasing sperm percentage with normal morphology so that the highest percentage of sperm with normal morphology was obtained in healthy rats receiving curcumin. However, Fe_2_O_3_ magnetic nanoparticles significantly reduced the percentage of sperm with normal morphology in the group of healthy rats and rats with scrotal hyperthermia. However, concomitant administration of Fe_2_O_3_ magnetic nanoparticles with curcumin improved sperm count with normal morphology. In general, the results showed that scrotal hyperthermia caused a sharp decrease in sperm percentage with normal morphology; however, administration of curcumin or Fe_2_O_3_ nanoparticles with curcumin greatly improved sperm percentage with normal morphology (Figure-4).

## Discussion

There have been studies in which scrotal hyperthermia-induced toxicity has been reported in mammals. The testicular function has been shown to be a temperature-dependent process, and scrotal hyperthermia can cause problems such as infertility [20]. Therefore, it is important to find solutions to reduce the effects of scrotal hyperthermia that have fewer side effects. In the present study, it was found that curcumin can improve sperm parameters in healthy rats and sperm parameters in scrotal hyperthermia rats. However, Fe_2_O_3_ magnetic nanoparticles reduced sperm parameters in healthy rats with scrotal hyperthermia. Nevertheless, when Fe_2_O_3_ magnetic nanoparticles were consumed with curcumin, sperm parameters showed a significant improvement. Curcumin has been shown to play its therapeutic role by acting as an antioxidant [21], and this has been attributed to the phenolic group in its molecular structure [22]. Lipid peroxidation has been shown to occur in scrotal hyperthermia, and curcumin reduces lipid peroxidation. Curcumin's sweeping activity against free radicals, including anionic superoxide and hydroxyl ions, has also been shown [22]. Therefore, the protective role of curcumin can be attributed to the sweeping of free radicals and their antioxidant activity. Therefore, in the present study, the improvement of sperm parameters by using curcumin can be attributed to the antioxidant properties of curcumin. This was demonstrated in another study conducted on mice [23], and the results of the current study are in line with the results of that study. Also, in the present study, it was shown that scrotal hyperthermia causes a sharp decrease in sperm parameters, especially in viability percentage. This can be attributed to the induction of apoptosis in testicular reproductive cells [5]. However, this damage from cell apoptosis was reduced by taking curcumin. Therefore, it can be stated that curcumin has anti-apoptotic effects on testicular cells in scrotal hyperthermia conditions. Therefore, curcumin can be a good option for protecting against infertility-induced environmental factors. In the present study, the negative effect of Fe_2_O_3_ magnetic nanoparticles on sperm parameters was observed in healthy and scrotal hyperthermia rats. The results of previous studies have also shown the toxic effects of nanoparticles on male reproductive cells [24,25]. The sensitivity of mammalian spermatogonial stem cells to nanoparticles has also been reported [26]. The mechanism of damage to reproductive cells is attributed to the induction of inflammation or edema in the interstitial tissue [27]. Oxidative damage due to nanoparticles and their reaction with cellular DNA and cell dysfunction has also been reported [28]. Cell death from nanoparticles has also been reported to be due to the autophagy mechanism [29]. Therefore, the toxic effects of Fe_2_O_3_ magnetic nanoparticles observed in the present study can be attributed to these mechanisms. However, co-administration of curcumin with Fe_2_O_3_ nanoparticles greatly improved sperm parameters, which can be attributed to the antioxidant properties of curcumin. It has also been shown that the mechanism of cell death due to nanoparticles is more likely to occur due to the autophagy mechanism [29]. Therefore, it can be stated that curcumin can improve sperm parameters by reducing the autophagy of sperm cells. However, more research is needed in this regard. In recent years, the green synthesis of nanoparticles by plants has attracted much attention and is being considered as an alternative to the chemical methods of nanoparticle synthesis [30]. Also, the green synthesis of nanoparticles is very affordable. Therefore, green synthesis of Fe_2_O_3_ nanoparticles and concomitant use of curcumin in patients with scrotal hyperthermia is recommended. The limitations of the present study were the difficulty of preparing animal models and nanoparticles under these conditions.

## Conclusion

The results of the current study suggested that curcumin in combination with Fe_2_O_3_ nanoparticles could greatly improve semen parameters and that these effects were attributed to their antioxidant properties. Therefore, it is suggested as a treatment option to reduce infertility caused by scrotal hyperthermia damage. The green synthesis of nanoparticles is also suggested in future studies.

## Acknowledgment

We would like to appreciate the cooperation of postgraduate studies and research deputy of North Tehran Branch of Azad University, Tehran, Iran, and the pathology department of Imam Khomeini Hospital, especially Ms. Hadiseh Mohammadpour and Dr. Parvaneh Naserzadeh from Beheshti School of Pharmacy for assisting to carry out the current project.

## Conflict of Interest

There are no conflicts of interest.

## References

[1] Garolla A, Torino M, Sartini B, Cosci I, Patassini C, Carraro U (2013). Seminal and molecular evidence that sauna exposure affects human spermatogenesis. Hum. Reprod.

[2] Pelliccione F, Micillo A, Cordeschi G, D’Angeli A, Necozione S, Gandini L (2011). Altered ultrastructure of mitochondrial membranes is strongly associated with unexplained asthenozoospermia. Fertil.

[3] Takahashi M (2012). Heat stress on reproductive function and fertility in mammals. Reprod. Med. Biol.

[4] Mieusset R, Bujan L, Mondinat C, Mansat A, Pontonnier F, Grandjean H (1987). Association of scrotal hyperthermia with impaired spermatogenesis in infertile men. Fertil.

[5] Rockett JC, Mapp FL, Garges JB, Luft JC, Mori C, Dix DJ (2001). Effects of hyperthermia on spermatogenesis, apoptosis, gene expression, and fertility in adult male mice. Biol. Reprod.

[6] Ruiz-Pesini E, Lapeña AC, Dı Íez C, Álvarez E, Enrı Íquez JA, López-Pérez MJ (2000). Seminal quality correlates with mitochondrial functionality. Clin. Chim. Acta.

[7] Jagetia GC (2007). Radioprotection and radiosensitization by curcumin.. The Molecular Targets and Therapeutic Uses of Curcumin in Health and Disease: Springe.

[8] Free M, Schluntz G, Jaffe R (1976). Respiratory gas tensions in tissues and fluids of the male rat reproductive tract. Biol. Reprod.

[9] Zangar RC, Davydov DR, Verma S (2004). Mechanisms that regulate production of reactive oxygen species by cytochrome P450. Toxicol. Appl. Pharmacol.

[10] Ahmadi F (2010). Effect of turmeric (Curcumin longa) powder on performance, oxidative stress state and some of blood parameters in broiler fed on diets containing aflatoxin B1. Glob. Vet.

[11] Khan RU, Naz S, Javdani M, Nikousefat Z, Selvaggi M, Tufarelli V (2012). The use of turmeric (Curcuma longa) in poultry feed. Poult. Sci. J.

[12] Aktas C, Kanter M, Erboga M, Ozturk S (2012). Anti-apoptotic effects of curcumin on cadmium-induced apoptosis in rat testes. Toxicol Ind Health.

[13] Sudjarwo SA, Giftania Wardani Sudjarwo K (2017). Protective effect of curcumin on lead acetate-induced testicular toxicity in Wistar rats. Res Pharm Sci.

[14] Sharaf H, Morsy F, Shaffie N, El-Shennawy A (2012). Histological and histochemical study on the protective effect of curcumin on ultraviolet irradiation induced testicular damage in albino rats. J Cytol Histol.

[15] Grynkiewicz G, Ślifirski P (2012). Curcumin and curcuminoids in quest for medicinal status. Acta Biochim. Pol.

[16] Tronc E, Ezzir A, Cherkaoui R, Chanéac C, Noguès M, Kachkachi H (2000). Surface-related properties of γ-Fe_2_O_3_ nanoparticles. J. Magn. Magn. Mater.

[17] Mahmoudi M, Sant S, Wang B, Laurent S, Sen T (2011). Superparamagnetic iron oxide nanoparticles (SPIONs): development, surface modification and applications in chemotherapy. Adv. Drug Deliv. Rev.

[18] Stephen ZR, Kievit FM, Zhang M (2011). Magnetite nanoparticles for medical MR imaging. Mater. Today Commun.

[19] Nasr-Esfahani MH, Aboutorabi R, Esfandiari E, Mardani M (2002). Sperm MTT viability assay: a new method for evaluation of human sperm viability. J. Assist. Reprod. Genet.

[20] Paul C, Teng S, Saunders PT (2009). A single, mild, transient scrotal heat stress causes hypoxia and oxidative stress in mouse testes, which induces germ cell death. Biol. Reprod.

[21] Piper JT, Singhal SS, Salameh MS, Torman RT, Awasthi YC, Awasthi S (1998). Mechanisms of anticarcinogenic properties of curcumin: the effect of curcumin on glutathione linked detoxification enzymes in rat liver. Int. J. Biochem.

[22] Reddy Acp, Lokesh BR (1994). Studies on the inhibitory effects of curcumin and eugenol on the formation of reactive oxygen species and the oxidation of ferrous iron. Mol. Cell. Biochem.

[23] Lin C, Shin D-G, Park SG, Chu SB, Gwon LW, Lee J-G (2015). Curcumin dosedependently improves spermatogenic disorders induced by scrotal heat stress in mice. Food Funct.

[24] Borm PJ, Kreyling W (2004). Toxicological hazards of inhaled nanoparticles-potential implications for drug delivery. J. Nanosci. Nanotechnol.

[25] Chen Y, Xue Z, Zheng  D , Xia K, Zhao Y, Liu T (2003). Sodium chloride modified silica nanoparticles as a non-viral vector with a high efficiency of DNA transfer into cells. Curr. Gene Ther.

[26] Braydich-Stolle L, Hussain S, Schlager JJ, Hofmann M-C (2005). In vitro cytotoxicity of nanoparticles in mammalian germline stem cells. Toxicol. Sci.

[27] Moridian M, Khorsandi L, Talebi A (2015). Morphometric and stereological assessment of the effects of zinc oxide nanoparticles on the mouse testicular tissue. Bratisl. Lek.

[28] Nel A, Xia T, Mädler L, Li N (2006). Toxic potential of materials at the nanolevel. Science.

[29] Duan J, Yu Y, Yu Y, Li Y, Wang J, Geng W (2014). Silica nanoparticles induce autophagy and endothelial dysfunction via the PI3K/Akt/mTOR signaling pathway. Int J Nanomedicine.

[30] Iravani S (2011). Green synthesis of metal nanoparticles using plants. Green Chem.

